# *Plasmodium falciparum*-Derived Uric Acid Precipitates Induce Maturation of Dendritic Cells

**DOI:** 10.1371/journal.pone.0055584

**Published:** 2013-02-06

**Authors:** Diana L. van de Hoef, Isabelle Coppens, Thomas Holowka, Choukri Ben Mamoun, OraLee Branch, Ana Rodriguez

**Affiliations:** 1 Division of Parasitology, Department of Microbiology, New York University School of Medicine, New York, New York, United State of America; 2 Department of Molecular Microbiology and Immunology, Johns Hopkins Malaria Research Institute, Baltimore, Maryland, United State of America; 3 Section of Infectious Disease and Department of Microbial Pathogenesis, Yale University School of Medicine, New Haven, Connecticut, United State of America; French National Centre for Scientific Research, France

## Abstract

Malaria is characterized by cyclical fevers and high levels of inflammation, and while an early inflammatory response contributes to parasite clearance, excessive and persistent inflammation can lead to severe forms of the disease. Here, we show that *Plasmodium falciparum*-infected erythrocytes contain uric acid precipitates in the cytoplasm of the parasitophorous vacuole, which are released when erythrocytes rupture. Uric acid precipitates are highly inflammatory molecules that are considered a danger signal for innate immunity and are the causative agent in gout. We determined that *P. falciparum-*derived uric acid precipitates induce maturation of human dendritic cells, increasing the expression of cell surface co-stimulatory molecules such as CD80 and CD86, while decreasing human leukocyte antigen-DR expression. In accordance with this, uric acid accounts for a significant proportion of the total stimulatory activity induced by parasite-infected erythrocytes. Moreover, the identification of uric acid precipitates in *P. falciparum*- and *P. vivax*-infected erythrocytes obtained directly from malaria patients underscores the *in vivo* and clinical relevance of our findings. Altogether, our data implicate uric acid precipitates as a potentially important contributor to the innate immune response to *Plasmodium* infection and may provide a novel target for adjunct therapies.

## Introduction

Malaria blood stage infection is characterized by cyclical fevers and induced inflammatory cytokinemia in the blood. Upon completion of the replication cycle in infected erythrocytes, *Plasmodium* merozoites are released and rapidly invade new erythrocytes to reinitiate a cycle of infection. In this process, the erythrocyte membrane, the parasitophorous vacuolar membrane and the parasite plasma membrane are each ruptured resulting in the concomitant release of merozoites and cellular contents into the bloodstream [1]. Because of the highly synchronous replication of parasites within erythrocytes during natural infections, the release of parasitic contents at the end of each cycle triggers an acute inflammatory response that causes the high cyclical fevers that associate with malaria. Excessive and persistent inflammation in *P. falciparum* infection contributes to severe malaria pathology and to the development of cerebral malaria and severe malarial anemia [2]–[4].

Three pathogen-associated molecular patterns (PAMPs) have been identified in *P. falciparum*: GPI-anchors [5], hemozoin [6], [7] and parasite DNA [8], [9]. Although their relative contribution to malaria-induced inflammation and pathology in patients remains unclear [10], [11], it appears that GPI-anchors alone cannot account for the innate immune stimulatory activity of malaria [12]. *Plasmodium* DNA bound to hemozoin [8], [9] or to parasite histones [13] induces the activation of TLR-9; however, direct activation of TLR-9 [14] or the Nlrp3 inflammasome [7], [15] by hemozoin has also been reported. In addition, *Plasmodium* AT-rich DNA is recognized by TLR9-independent immune pathways that induce type I IFN expression [9].

The inflammatory properties of uric acid have been recognized for several decades due to its pathological role in gout, a disease caused by an inflammatory response to uric acid crystallization in joint synovial fluid [16]. Uric acid was also identified as an endogenous danger-associated molecular pattern (DAMP) released from injured or dying cells that significantly contributes to cell death-induced inflammatory responses *in vivo* [17]–[19]. Furthermore, as a potent adjuvant, uric acid stimulates the maturation of dendritic cells and increases expression of co-stimulatory molecules that prime T-cell responses [20].

Here, we show that uric acid precipitates accumulate within *P. falciparum*-infected erythrocytes. Utilizing methods that allow direct visualization and measurement of precipitated uric acid in infected erythrocytes, we demonstrate that uric acid precipitates arise within the parasitophorous vacuole of replicating *P. falciparum*, and they are subsequently released upon rupture of infected erythrocytes. These precipitates induce the maturation of human dendritic cells *in vitro*, and this response is significantly inhibited when uric acid is depleted. Our findings suggest that uric acid precipitates may be important contributors to the innate immune response induced by the rupture of infected erythrocytes. In this way, an evolutionarily conserved DAMP such as uric acid may also function as a PAMP in the host innate immune response to malaria.

We have previously shown that *Plasmodium*-infected erythrocytes accumulate hypoxanthine, which is a precursor for uric acid synthesis. Upon release from ruptured erythrocytes, hypoxanthine is degraded into uric acid, which can become inflammatory [21], [22]. Our current study shows that uric acid precipitates accumulate within infected erythrocytes, uncovering an additional and more relevant mechanism for the activation of innate immunity in malaria. We also established that in natural malaria infections, precipitates of uric acid accumulate within *P. falciparum*- and *P. vivax*-infected erythrocytes. These results highlight the potential clinical importance of parasite-derived uric acid precipitates, which may provide a new target for much needed adjunct therapies in the treatment of malaria.

## Results

### Uric Acid Precipitates are Found in *P. falciparum*-infected Erythrocytes

**Figure 1:**
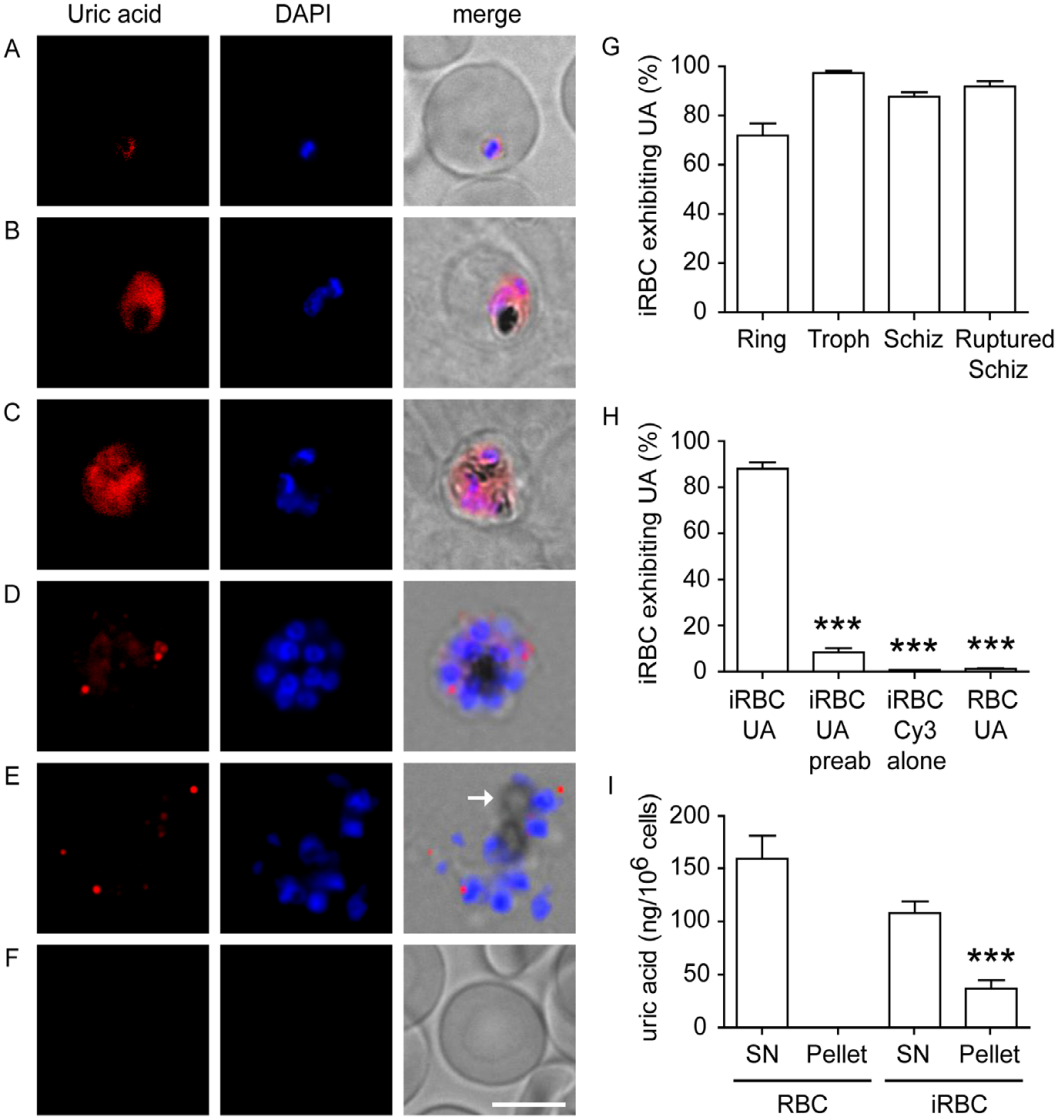
Uric acid precipitates accumulate within *P. falciparum*-infected red blood cells *in vitro*. Uric acid immunostaining (red) and DAPI labeling of nuclei (blue) of *P. falciparum*-infected red blood cells at different stages of development: ring (A), trophozoite (B and C), schizont (D) and merozoite (ruptured schizont with free merozoites) (E). Uninfected control red blood cells (F) are shown. Merged images with phase contrast are shown in the right column. Hemozoin can be observed as intracellular dark areas in B – E (a representative hemozoin crystal is marked by an arrow in panel E). Bar is 5 µm. (G) Quantitation of infected erythrocytes (iRBC) that exhibit uric acid immunostaining at different stages of development (Troph, Trophozoites; Schiz, schizonts, as shown in panels D and E). Data are displayed as mean ± SEM (n = 100 individual iRBC quantified from three independent experiments). (H) *P. falciparum*-infected schizonts incubated with anti-uric acid antibodies preabsorbed with uric acid crystals (iRBC UA preab) showed low levels of uric acid immunolocalization, compared to iRBC labeled with unprocessed anti-uric acid antibodies (iRBC UA). Staining with secondary antibodies alone (iRBC Cy3 alone) was used as a control. Data are displayed as mean ± SEM (n = 200 individual iRBCs or RBCs quantified from three independent experiments; ***p<0.001 relative to iRBC UA). (I) Quantitation of uric acid levels in supernatant (SN) and pellet fractions from lysates of purified *P. falciparum*-infected schizonts (iRBC) or uninfected erythrocytes (RBC). A colorimetric absorbance assay was used to determine uric acid levels. Data are displayed as mean ± SEM (n = 3 independent experiments; ***p<0.001 relative to control RBC pellet).

To determine if uric acid precipitates accumulate in *P. falciparum*-infected erythrocytes, we initially performed immunofluorescence analysis of parasites cultured *in vitro* using rabbit polyclonal antibodies that specifically stain precipitated, and not free, uric acid. We observed a diffuse uric acid staining pattern in ring and trophozoite stage parasites (Fig. 1A–C) consistent with a cytoplasmic distribution, while a more distinct punctate pattern was observed in schizonts (Fig. 1D). In ruptured schizonts, relatively small uric acid-positive aggregates were identified extracellularly in close proximity to free merozoites and hemozoin (Fig. 1E). As a control, we stained uninfected erythrocytes, which did not display detectable levels of uric acid aggregates (Fig. 1F). Quantitation of purified infected erythrocytes labeled with anti-uric acid antibodies revealed that approximately 85% of mid- to late-stage infected erythrocytes exhibit punctate uric acid staining (Fig. 1G).

To confirm antibody specificity, anti-uric acid antibodies were preabsorbed with synthetic uric acid crystals prior to labeling. The uric acid precipitate signal was reduced by >80% in *P. falciparum*-infected erythrocytes following antibody preabsorption, compared to iRBCs labeled with unprocessed antibodies (Fig. 1H). The specificity of the preabsorption process was confirmed by incubating rabbit anti-Giantin antibodies, which label the Golgi complex in infected erythrocytes, with synthetic uric acid crystals. This treatment did not alter antibody reactivity, as assessed by immunofluorescence (Fig. S1). Furthermore, a similar punctate uric acid staining pattern in infected erythrocytes was observed using a second, independently produced anti-uric acid antibody (Fig. S2).

To confirm that uric acid precipitates accumulate in infected erythrocytes, we used ultracentrifugation of lysates derived from infected erythrocytes to separate soluble uric acid from the precipitated form. Uric acid quantification was performed using a colorimetric assay, which is dependent on the ability of uric acid to bind to iron ions [23]. In both infected and uninfected erythrocyte lysates, soluble uric acid was detected in the supernatant fraction (Fig. 1I), consistent with uric acid being present in the cytoplasm of erythrocytes [24]. Notably, a significant amount of uric acid was detected in the pellet fraction of infected erythrocytes but not in uninfected erythrocytes (Fig. 1I), indicating the presence of precipitated uric acid within *P. falciparum*-infected erythrocytes.

**Figure 2:**
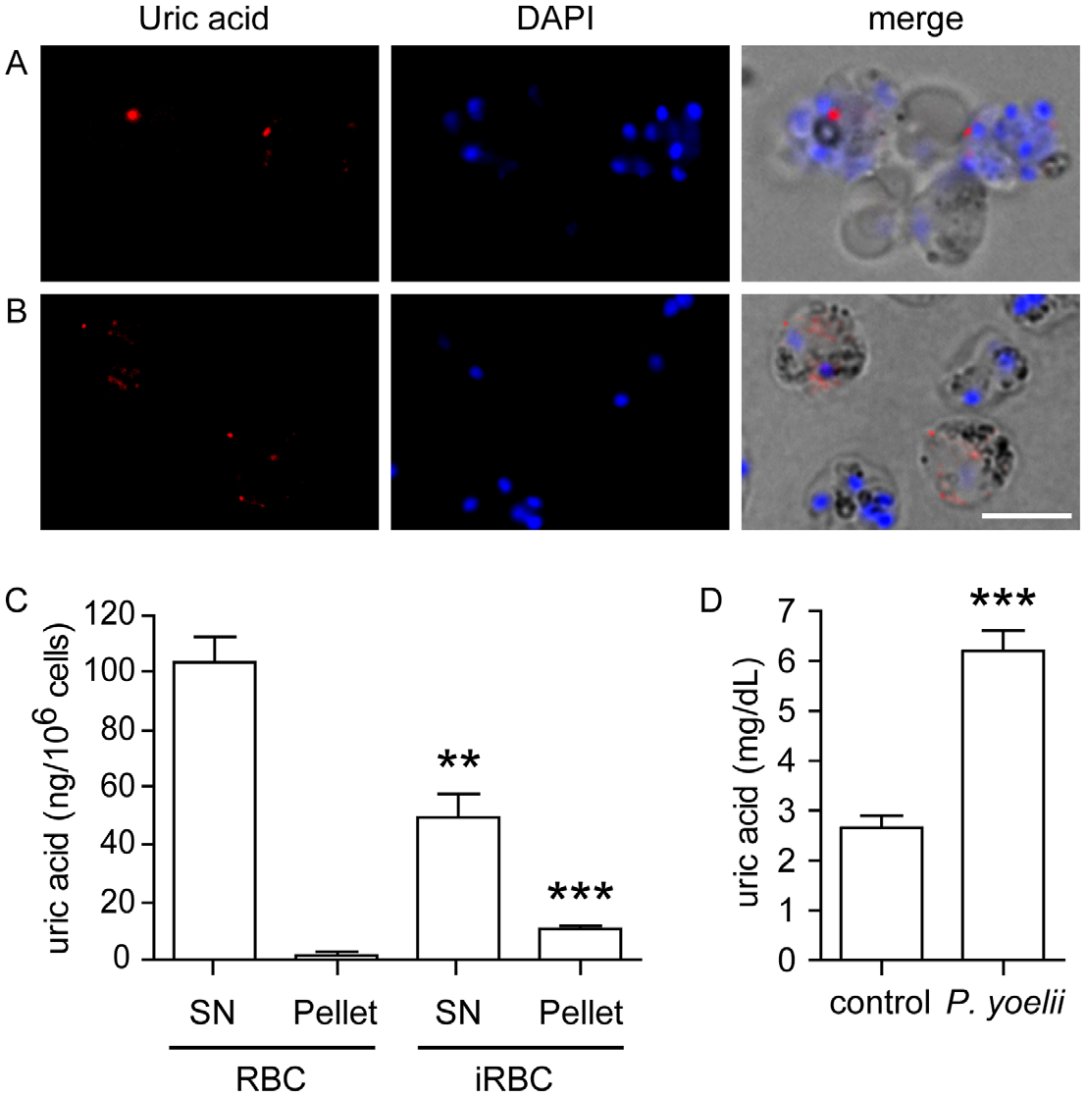
Uric acid precipitates accumulate in murine *Plasmodium*-infected red blood cells *in vivo*. Purified *P. yoelii* (A) and *P. berghei* (B) schizonts from infected mice, labeled with anti-uric acid antibodies, show characteristic uric acid immunostaining patterns (red). DAPI (blue) labels nuclei. Bar is 5 µm. (C) Quantitation of uric acid levels in supernatant (SN) and pellet fractions of lysates of purified *P. yoelii* schizonts (iRBC) or control uninfected erythrocytes (RBC). (D) Quantitation of uric acid plasma levels, using a colorimetric absorbance assay, in uninfected control and *P. yoelii*-infected mice at day 9 of infection. Data in C and D are displayed as mean ± SEM (n = 6 control and n = 6 *P*. *yoelii*-infected mice in each experiment; **p<0.01, ***p<0.001 relative to control).

To investigate the occurrence of uric acid precipitates within erythrocytes *in vivo*, we isolated *Plasmodium*-infected erythrocytes from *P yoelii*- and *P*. *berghei*-infected mice. Immunostaining of these erythrocytes revealed the presence of uric acid-positive puncta, similar to those observed in our *in vitro* studies (Fig. 2A and B). Uric acid precipitates were also detected in the pellet fraction from lysates of *P*. *yoelii*-infected erythrocytes (Fig. 2C). Furthermore, levels of plasma uric acid were elevated in *P yoelii*-infected mice at day nine of infection, relative to control plasma (Fig. 2D), which is consistent with what has been observed in *P*. *falciparum*- and *P*. *vivax*-infected patients [25]–[28].

### Uric Acid Precipitates are Found in the Cytoplasm of *P. falciparum* within Infected Erythrocytes

**Figure 3:**
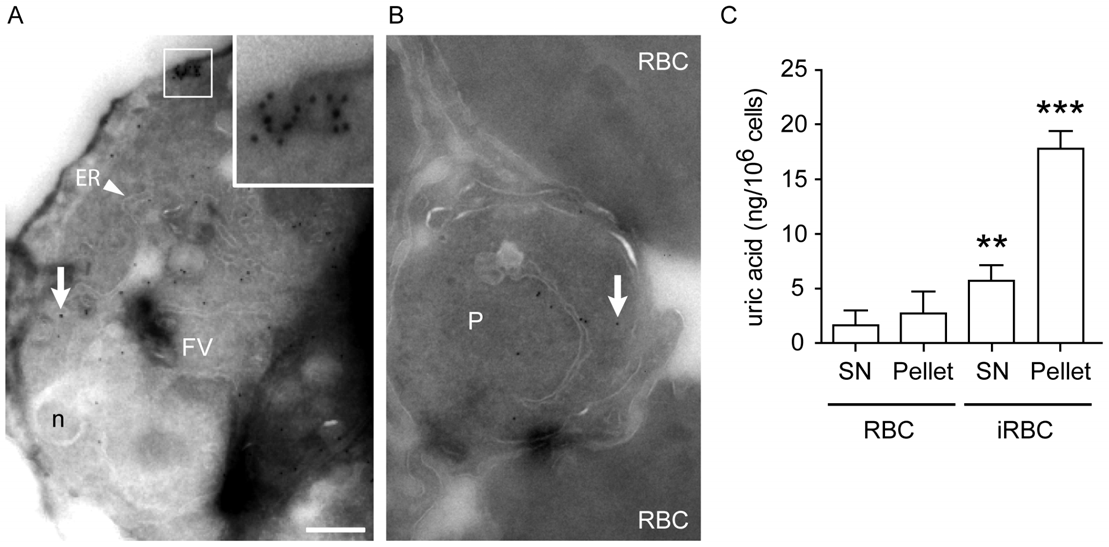
Immunolocalization of uric acid in *P. falciparum* cytoplasm. (A and B) Immunogold staining of intravacuolar *P. falciparum* demonstrates cytoplasmic distribution of precipitated uric acid. Arrows mark representative uric acid precipitates in the cytoplasm of iRBC. Aggregates of uric acid are also detected at the parasite periphery (A, inset). One hundred sections of labeled parasites, which included >70 parasitophorous vacuoles, were analyzed. RBC, uninfected erythrocytes; ER, endoplasmic reticulum; FV, food vacuole; n, nucleus. Bar is 200 nm. (C) Purified intact parasitophorous vacuoles were obtained from infected erythrocytes at the schizont stage (iRBC) using saponin treatment. After low-speed centrifugation, the purified parasite fraction was collected as a pellet. Pellets were lysed and ultracentrifugation was performed to obtain supernatant (SN) and pellet fractions. Uninfected erythrocyte (RBC) lysates were processed in parallel. Quantitation of uric acid was performed using a colorimetric absorbance assay. Data are displayed as mean ± SEM (n = 3 independent experiments; **p<0.01, ***p<0.001 relative to RBC controls).

To determine the precise origin of the uric acid precipitates, fine localization of uric acid within infected erythrocytes was performed by immunoelectron microscopy using anti-uric acid antibodies coupled to 10 nm gold particles. This analysis revealed the presence of uric acid precipitates in the cytoplasm of *P. falciparum* within infected erythrocytes, with negligible labeling of uninfected erythrocytes (Fig. 3A and B). Staining was specifically observed in the parasitophorous vacuole of *P. falciparum*, and not in the food vacuole or other organelles.

To further confirm the cytoplasmic localization of uric acid, *P*. *falciparum*-infected erythrocytes were treated with saponin to separate intact parasites within the parasitophorous vacuole from the surrounding erythrocyte plasma membrane, as previously described [29]–[31]. Higher levels of uric acid were detected in the pellet fraction of lysates derived from purified intact parasites, compared to the supernatant fraction and to control RBCs, indicating that uric acid is mostly found in precipitated form within the parasite cytoplasm (Fig. 3C).

### Uric Acid Precipitate Formation is Dependent on Extracellular Hypoxanthine

**Figure 4:**
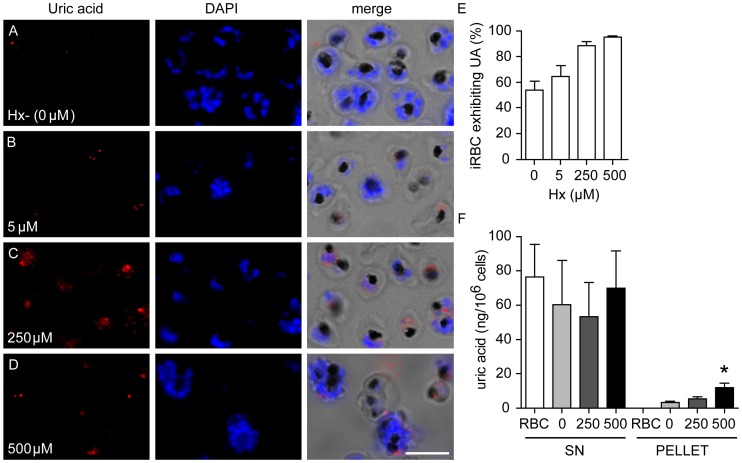
Formation of uric acid precipitates in *P. falciparum*-infected erythrocytes is dependent on exogenous hypoxanthine. *P. falciparum*-infected erythrocytes were cultured in hypoxanthine free (A) and hypoxanthine-supplemented media as the unique purine source (B, 5 µM; C, 250 µM, D, 500 µM). Uric acid antibodies label precipitates within infected parasites *in vitro*. DAPI labels nuclei (blue). Bar is 5 µm. (E) Quantitation of infected erythrocytes that are positive for uric acid immunostaining. Data are displayed as mean ± SEM (n>100 schizonts in duplicate, from three independent cultures). (F) Quantitation of uric acid in lysate fractions of purified *P. falciparum* schizonts cultured in increasing hypoxanthine concentrations: Hx- [0 µM Hx] (light grey), 250 µM Hx (dark grey) and 500 µM Hx (black). Ultracentrifugation was performed on lysates to obtain supernatant (SN) and pellet fractions. Uninfected erythrocytes (RBC; white) were processed in parallel. Quantitation of uric acid using a colorimetric absorbance assay is shown. Data are displayed as mean ± SEM (n = 3 independent cultures; *p<0.05 relative to Hx- [0 µM Hx] pellet control).

Hypoxanthine is a metabolic precursor of uric acid and is essential for *Plasmodium* growth [32]. Its transport inside the parasite involves a low affinity, high capacity transporter, PfNT1 [33], [34]. We therefore tested whether extracellular hypoxanthine affected the formation of uric acid precipitates in infected erythrocytes. Immunofluorescence analyses revealed significant reductions in levels of intracellular uric acid precipitates when hypoxanthine concentrations in the culture media were limiting. The punctate uric acid staining appeared less frequently and with lower intensity in cells grown in low hypoxanthine media (Fig. 4, compare panels A and B with panels C and D). Parasites grown in low exogenous hypoxanthine conditions show few, low intensity uric acid precipitates in approximately 50% of the cells in culture, while over 80% of infected erythrocytes in high hypoxanthine conditions show strong uric acid immunostaining (Fig. 4E). We also observed a significant decrease in the levels of precipitated uric acid in lysates derived from infected erythrocytes cultured under low hypoxanthine conditions relative to those grown in high hypoxanthine levels (Fig. 4F). The total uric acid concentration (SN plus pellet) appears unmodified by the presence or absence of hypoxanthine in the medium, suggesting that there are no major increases in uric acid concentration from hypoxanthine degradation and that uric acid precipitation is possibly induced by alternative mechanisms. It is important to note that when exogenous hypoxanthine was not added to culture media, we still detect low levels of hypoxanthine in culture supernatants (Fig. S3), which is likely derived from the spontaneous lysis of some erythrocytes present in the culture [35]. We found that this low concentration of hypoxanthine was sufficient to support parasite growth for a 48 h replication cycle.

### Uric Acid Precipitates Induce Maturation of Human Dendritic Cells

The activation of immune cells by uric acid has been well-documented, particularly in gout [16]. To determine whether uric acid derived from *P. falciparum*-infected erythrocytes could stimulate an innate response in immune cells, human dendritic cells from peripheral blood were stimulated with different fractions of *P. falciparum*-infected erythrocytes.

**Figure 5:**
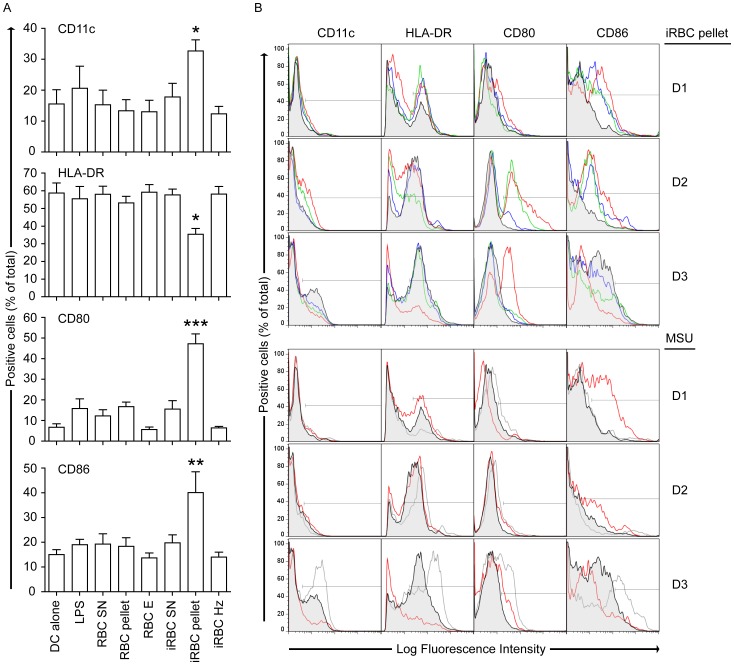
Uric acid precipitates from purified *P. falciparum* schizonts induce maturation of dendritic cells. (A) Dendritic cells enriched from human peripheral blood mononuclear cells were co-cultured with *P. falciparum* lysate fractions for 42 h. RBC fractions were used as control. Surface expression levels of CD11c, CD80, CD86 and HLA-DR were analyzed in dendritic cells gated for HLA-DR+. *P. falciparum* pellet fractions (iRBC pellet) induce significantly high CD11c, CD80 and CD86 surface marker expression levels, and reduced HLA-DR expression, relative to dendritic cells alone and to RBC fractions (n = 7 independent human blood donors and n = 6 independent iRBC lysates; *p<0.05; **p<0.01; ***p<0.001, one-way ANOVA with Fisher’s post hoc test). DC, dendritic cells; E, Eluate; Hz, hemozoin; LPS, lipopolysaccharides; SN, supernatant. (B) Dendritic cells from 3 independent donors (D1–3) were co-cultured with different fractions. *Top panels*: dendritic cells alone (grey filled lines) and co-cultured with lysates of purified iRBC pellet (red), iRBC pellet treated with DNase (green) and iRBC pellet from cultures grown in low exogenous hypoxanthine conditions and treated with uricase (blue). *Bottom panels*: dendritic cells co-cultured with LPS (light grey) and MSU (red). Control dendritic cells alone (grey filled lines) show baseline expression levels, similar to dendritic cells co-cultured with fractions derived from control uninfected erythrocytes (data not shown). Shown are results from three independent donors of peripheral blood co-cultured with three independent iRBC pellet fractions and two independent iRBC Hx- (0 µM exogenous hypoxanthine) pellet fractions.

Infected and uninfected erythrocyte lysates were fractionated by ultracentrifugation as described above to separate the supernatant from the pellet fraction, which contains precipitated uric acid. The pellet fraction was further processed using a magnetic column to remove hemozoin, as previously described [36]. Human dendritic cells enriched from whole peripheral blood mononuclear cells were co-cultured with the lysate fractions for 42 h, a previously established time-frame that is compatible with dendritic cell maturation in response to *Plasmodium* [37]. Dendritic cells normally upregulate surface expression levels of co-stimulatory molecules (CD80, CD86) and major histocompatibility complex class II molecules (human leukocyte antigen [HLA] in humans) upon exposure to a maturation stimulus [37], [38]. To assess cell maturation in response to *P*. *falciparum* fractions, we measured the surface expression levels of CD80, CD86 and HLA-DR, in addition to CD11c, a surface marker of myeloid dendritic cells that is normally not upregulated upon maturation [39]. As a control, dendritic cells were incubated with lysate fractions from uninfected erythrocytes. In seven independent human donors tested, we found elevated levels of CD11c, CD80 and CD86 surface expression in dendritic cells in response to iRBC pellet fractions, which contain uric acid precipitates, compared to dendritic cells incubated with supernatant or hemozoin fractions, or with control RBC fractions (Fig. 5A). These results indicate that the most potent stimulatory activity of *P. falciparum*-infected erythrocytes is found in the pellet fraction and is not associated with hemozoin, as this fraction failed to induce maturation of dendritic cells. The *P. falciparum* lysate pellet fraction caused significantly higher induction of human dendritic cell maturation than LPS, which is routinely used as a reference for maturation.

The surface expression of HLA-DR normally increases upon stimulation of dendritic cells [38], and interestingly, we show a marked decrease in HLA-DR expression levels after incubation with *P. falciparum* pellet fractions (Fig. 5A). Dendritic cells isolated from peripheral blood obtained from malaria patients in Kenya show similarly low levels of HLA-DR surface expression, as compared to healthy volunteers [40]. Together, this suggests that dendritic cells may be functionally impaired in malaria infection.

To determine the importance of uric acid precipitates in *P. falciparum*-infected erythrocytes, we fractionated lysates from *P. falciparum*-infected erythrocytes grown in low concentrations of hypoxanthine. As described above, these parasites contain significantly lower levels of uric acid precipitates. We treated these lysate fractions with uricase, an enzyme that degrades uric acid even in precipitated form [41], [42]. Parallel samples with lysate fractions from *P. falciparum*-infected erythrocytes grown in high hypoxanthine levels were used. *Plasmodium* DNA has been identified as a strong activator of dendritic cell maturation [8], [9], [12], [13], therefore we also prepared pellet fractions treated with DNase. The overall response of dendritic cells from three independent human donors (D1, D2 and D3) to the treated fractions was analyzed (Fig. 5B, top panels). Despite the expected variation between donors, pellet fractions with low levels of uric acid precipitates (low hypoxanthine+uricase treatment) were less stimulatory to human dendritic cells (Fig. 5B, top panels, blue), as compared to mock-treated pellet fractions from parasites grown in high concentrations of hypoxanthine (Fig. 5B, top panels, red). In particular, in response to the pellet fractions from parasites grown under high hypoxanthine conditions, the surface expression of CD80 was significantly increased in all three donors and CD86 expression increased in two out of the three donors. To confirm the stimulatory effect of *P. falciparum*-derived uric acid on dendritic cells, we treated iRBC pellet fractions (high hypoxanthine) with uricase, and observed a significant reduction in the maturation of dendritic cells from four additional human donors, compared to mock-treated iRBC pellet fractions (Fig. S4). As control, uricase treatment of RBC pellet fractions did not induce significant effects on dendritic cell maturation, indicating that uricase did not carry contaminants that could be stimulatory, such as LPS (Fig. S4). We additionally found that pellets treated with DNase induced low surface co-stimulatory expression levels (Fig. 5B, top panels, green), indicating that both uric acid precipitates and DNA are important contributors to the maturation of dendritic cells in response to *P. falciparum*.

Dendritic cells obtained from the same donors were induced by LPS and monosodium urate crystals (MSU). It was previously reported that dendritic cells respond differently to MSU and LPS *in vitro* [20]. In human dendritic cells from D1 and D2, CD86 expression levels increased in response to MSU (Fig. 5B, bottom panels, red), compared to control (filled grey) and LPS (light grey lines). CD80 expression levels increased in D2 and D3 in response to MSU stimulation; however, MSU stimulation in D3 reduced the surface expression levels of CD11c, HLA-DR and CD86 (Fig. 5B, bottom panels, red). Interestingly, *P. falciparum* lysate pellet fractions induced similar expression profiles to those observed with MSU in each donor (Fig. 5B; compare both panels). Control RBC lysate fractions treated under similar conditions with DNase did not induce maturation of dendritic cells (data not shown).

### Uric Acid Precipitates Accumulate in *P. falciparum and P. vivax*-infected Blood Cells in Malaria Patients

**Figure 6:**
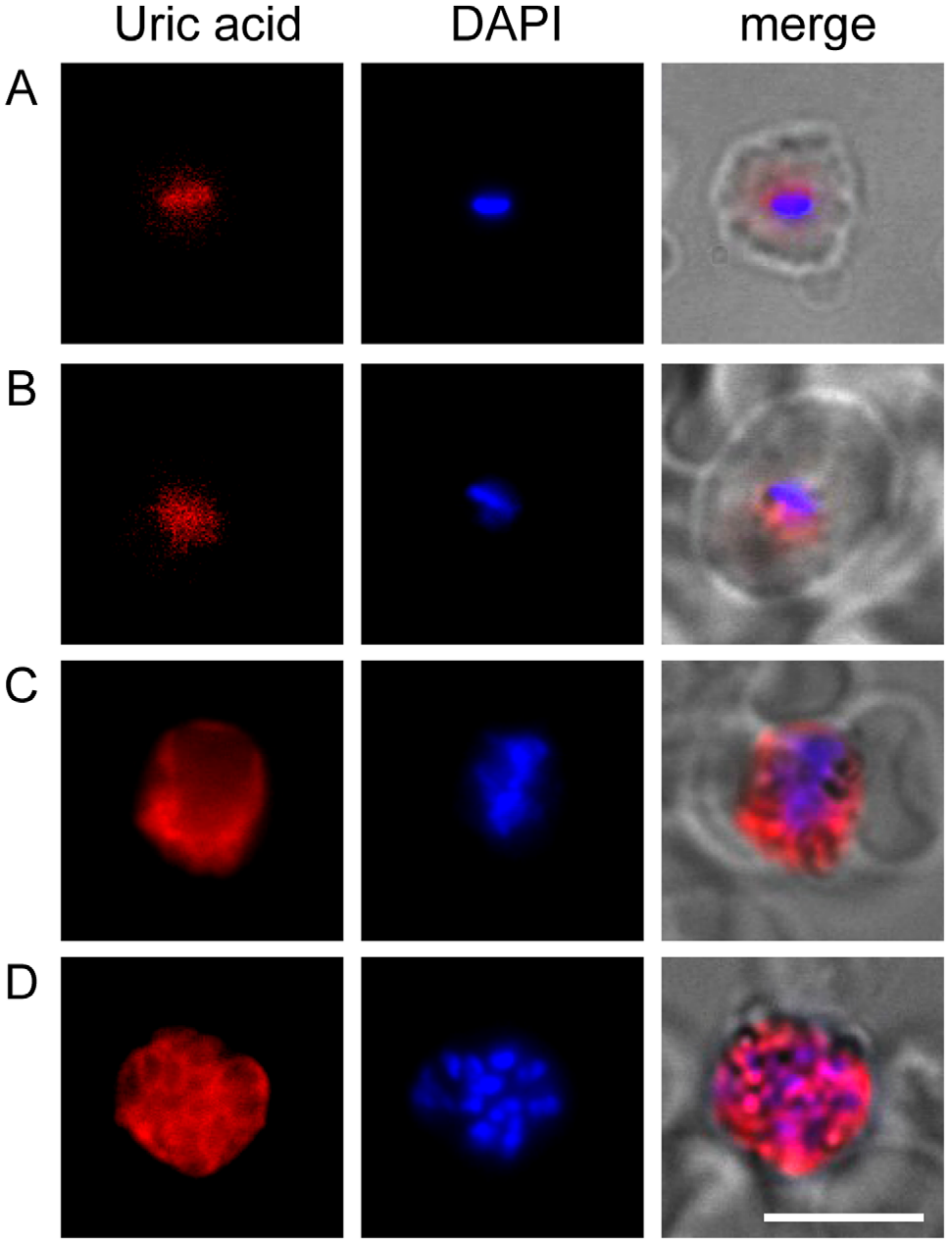
Blood-stage parasites from human malaria patients in the Peruvian Amazon show accumulation of UA precipitates. Blood samples from patients infected with *P. falciparum* (A) or *P. vivax* (B–D) exhibit uric acid immunostaining (red), and DAPI labeling of parasite nuclei (blue). Representative examples of rings (A, B), trophozoites (C) and schizonts (D) are shown. Bar is 5 µm.

We next sought to determine whether uric acid accumulates in *Plasmodium* parasites obtained from patients in malaria endemic regions. To do this, we collected blood samples from malaria patients residing in several communities in the low transmission Peruvian Amazon [43]. In all *P. falciparum* samples collected (n = 8 patients; 0.05–2% parasitemia in thin blood smears), uric acid precipitates were detected in infected erythrocytes. Uric acid immunolocalization showed a diffuse staining pattern in *P. falciparum* rings (Fig. 6A), similar to our observations of iRBC rings grown *in vitro* (Fig. 1A). *P. falciparum* trophozoites and schizonts were not observed as these do not circulate in peripheral blood. Gametocytes were also absent in these samples.

In *P. vivax* samples (n = 10 patients; 0.1–1% parasitemia in thin blood smears), we found diffuse uric acid localization in rings (Fig. 6B), trophozoites (Fig. 6C) and schizonts (Fig. 6D). Close observation of *P. vivax* schizonts indicates that uric acid staining localizes to the parasitophorous vacuole outside of individual merozoites. This is similar to the cytoplasmic localization of uric acid within cultured *P. falciparum* parasites (Fig. 1D). Although the percentage parasitemia differed for each patient, we found that all of the *P. falciparum* and *P. vivax* samples exhibited uric acid staining in a high proportion (>83%) of infected erythrocytes.

## Discussion

We found that the pellet fraction of *P. falciparum*-infected erythrocytes, containing precipitates of uric acid, strongly induced maturation of dendritic cells, and this response was significantly reduced when uric acid levels were depleted. Uric acid is a strong adjuvant and a proinflammatory danger-associated molecular pattern (DAMP) that alerts the immune system of cellular damage [19], [20], [44], and this involves a precipitated, not soluble, form of uric acid [20], compatible with our findings. Uric acid crystals also have a well-known causative role in gout, as elevated plasma uric acid levels promote crystallization in joint synovial fluid, inducing a strong inflammatory response [45], [46].

The mechanism of uric acid precipitation in *Plasmodium* is still unknown. The total levels of uric acid (soluble and precipitates) are similar in both *P*. *falciparum*-infected and uninfected erythrocytes, and so it is unlikely that increased intracellular levels of uric acid cause precipitation within the cytoplasm of *P. falciparum*. Acidic pH and low temperature are two key factors that trigger precipitation of uric acid, but these conditions are generally not found within *P. falciparum*-infected erythrocytes [47], excluding the highly acidic food vacuole [48] that was found to be devoid of uric acid precipitates. A possible cause for the formation of uric acid precipitates could involve increased levels of related molecules like hypoxanthine, as this would decrease the solubility of uric acid. Our data is consistent with this hypothesis, as increasing concentrations of extracellular hypoxanthine resulted in higher uric acid precipitation in the parasite without affecting total levels of intracellular uric acid. The presence of specific proteins and lipids in the parasite cytoplasm may serve as nucleating factors for uric acid precipitation, similar to what occurs in calcium stone and gallstone formation [49]–[51]; however, further studies are needed to determine the specific mechanisms involved in uric acid precipitation within *Plasmodium* parasites.

Uric acid is a physiological byproduct of nucleic acid metabolism and is normally present as a soluble monosodium urate ion in blood serum. In most mammals, the liver enzyme uricase degrades plasma uric acid to allantoin, which is further degraded to urea and excreted [41]. Humans and most primates lack uricase activity because of a mutation affecting the function of uricase, and consequently uric acid plasma levels are elevated compared to other mammals [18], [52]–[54]. The plasma uric acid concentration in humans, approximately 7 mg/dL, lies near the solubility limit and this creates a near perfect environment for precipitate formation [52]. The absence of plasma uricase activity may therefore contribute to malaria inflammation, as humans would have difficulty eliminating highly inflammatory uric acid precipitates released by ruptured *Plasmodium*-infected erythrocytes. Indeed, elevated plasma urate levels were found in *Plasmodium* infections in mice [55] and in humans, which correlate with disease severity in *P. falciparum* [25]–[27] and with parasitemia in *P. vivax* [28]. In addition, a correlation between uric acid plasma levels and inflammatory cytokines in children infected with *P. falciparum* in Mali was found [56]. Together, these data suggest that plasma urate levels may represent a possible biomarker that indicates disease severity. Although elevated uric acid levels in the blood can be a consequence of kidney damage that often associates with severe malaria [57], it is likely that release of both hypoxanthine [21], [22] and precipitates of uric acid from *Plasmodium*-infected erythrocytes contributes to elevated plasma uric acid levels in malaria.

In a clinical study of *P. falciparum*-infected patients with acute complicated malaria, patients treated with the anti-malarial drug, quinine, in combination with allopurinol, an inhibitor of uric acid synthesis, showed a faster decrease in fever and splenomegaly compared to patients treated with quinine alone, even though parasite levels decreased at a similar rate in both groups [58]. This suggests that uric acid plays an important role in the inflammatory response to *P. falciparum* infection in humans, which is highly significant, particularly because a long-term effort in malaria research has been to identify parasite-derived inflammatory molecules that can be targeted early during infection.

It is generally accepted that excessive and persistent inflammation contributes to the most severe form of malaria, cerebral malaria [2], [4]. Despite the rapid administration of anti-parasitic treatments following cerebral malaria diagnosis, 20% of children will succumb to this syndrome [4]. In these circumstances, clinicians advocate for adjunct anti-inflammatory therapies to help alleviate cognitive impairment and prevent death, while anti-parasitic therapies eliminate circulating *Plasmodium* parasites [4], [59].

Three *Plasmodium* PAMPs that have been described include GPI anchors, DNA and hemozoin. GPI anchors activate TLR2 and to a minor extent TLR4 [60], while parasite hemozoin has been described to activate TLR9 [6], [14], in addition to activating the Nlrp3 inflammasome [7], [15], [61]. Other groups have shown hemozoin to be inert [8], [12]. Parasite DNA was found to activate TLR9 either bound to hemozoin [8] or to parasite histones [13]. Additionally, parasite AT-rich DNA can induce a TLR9-independent pathway [9]. It seems likely that differences in the lysis conditions and in the purification of hemozoin from infected erythrocytes could affect the characteristics of hemozoin or the types of molecules that are bound to it, and this may contribute to the observed differences in its stimulatory capacity. One report suggested that uric acid was a mediator of the hemozoin-induced response [7]; however, this is independent from our study as the mice were injected with synthetic hemozoin without the presence of *Plasmodium*-derived uric acid.

Studies in mice indicate that the inflammatory response to *Plasmodium* infection and the host susceptibility to experimental cerebral malaria are independent of Nlrp3 inflammasome-dependent caspase-1 activation of IL-1β and IL-18 [62]. Nlrp3 can however influence experimental cerebral malaria independently of caspase-1 and IL-1β [62], [63], suggesting a non-classical role for Nlrp3 in malaria immunopathology. Other studies showed that the inflammasome was not involved in the adjuvant effect of whole *P. falciparum* lysates or synthetic hemozoin when injected into mice [14], nor in the immunomodulatory effect of caspase-12 [64]. It is important to remark that these studies regarding the role of the inflammasome in malaria have been performed in mice, which have strong plasma uricase activity that degrades *Plasmodium*-derived uric acid, which may limit its inflammatory activity. The few studies performed in humans regarding uric acid in malaria [56], [58], including the clinical study in patients treated with quinine and allopurinol that was mentioned above, suggest that uric acid plays an important role in malaria-induced inflammation in humans.

Antigen presenting cells, in particular dendritic cells, express HLA-DR, adhesion molecules and co-stimulatory molecules, including CD80 and CD86, in response to maturation stimuli [38]. We found a significant increase in dendritic cell surface expression of CD80 and CD86 in response to pellet fractions of *P*. *falciparum* lysates after removal of hemozoin. We presented a detailed analysis of *P. falciparum* fractions using dendritic cells isolated from seven different human donors, and believe these results to be highly significant because previous studies frequently used mouse cells as responders to *P. falciparum* lysates [12], [13]. Regardless of the expected variation in dendritic cell responses from different blood donors, a consistent decrease in the maturation of these cells was observed when pellet fractions were depleted of uric acid or DNA, indicating a major role for both uric acid precipitates and parasite DNA in the immune response to *P. falciparum*.

Several mechanisms have been proposed to explain how pathogens subvert host immunity, including impairment of dendritic cells during malaria infection. Reduced HLA-DR expression has been observed in human malaria patients and mouse models of malaria, and this can affect both antigen presentation in dendritic cells and T cell activation [40], [65]–[68]. Importantly, HLA class I and class II polymorphisms were shown to protect against severe malaria infection in West African children [69]. We observed a striking reduction in HLA-DR expression in response to *P. falciparum*-derived uric acid precipitates and DNA, suggesting that these factors may be involved in suppression of human dendritic cell functions. Further investigation is needed to determine the mechanisms involved in the dendritic cell response to *P*. *falciparum*-derived uric acid and nucleic acids.

The activation of innate immunity in malaria provides an important balance between parasite survival and host defense. The danger signal uric acid triggers a high inflammatory response, particularly in humans and primates that lack uricase activity. The strong dendritic cell response to *P. falciparum-*derived uric acid precipitates *in vitro* suggest that these precipitates could offer a novel molecular target for anti-inflammatory therapies in malaria.

## Materials and Methods

### Ethics Statement

This study had ethical approval from New York University Institutional Review Board (number 08-982) and from the Peruvian Ministry of Health Ethical Review Committee. All participants and/or their legal guardians provided written informed consent and/or assent, and these approvals continue with annual review. This study was carried out in strict accordance with the recommendations in the Guide for the Care and Use of Laboratory Animals of the National Institutes of Health. The protocol was approved by the Institutional Animal Care and Use Committee of New York University School of Medicine, which is fully accredited by the Association for Assessment and Accreditation of Laboratory Animal Care International (AAALAC).

### Parasite Culture

Erythrocyte asexual stage cultures of the *P. falciparum* strain 3D7 were maintained in culture medium (RPMI 1640, 25 mM HEPES, 10 ug/mL gentamycin, 250 µM hypoxanthine, pH 6.75), supplemented with 25 mM sodium bicarbonate and 0.5% Albumax II. A 5% hematocrit (O positive, sickle cell negative, leukocyte depleted erythrocytes) was maintained in the atmospheric conditions of 1% oxygen, 5% carbon dioxide and 94% nitrogen. The culture medium was changed daily and the cultures were sub-cultured to maintain a parasitemia of less than 6%. Parasite cultures were kept synchronized using gelatin flotation. Cultures were routinely tested for mycoplasma contamination using MycoAlert Mycoplasma Detection Kit (Lonza) and were never found to be positive. To determine parasitemia, the number of parasitized erythrocytes from 500 cells in a Giemsa stained blood smear were counted. *P. yoelii* 17X NL and *P. berghei* ANKA were used to infect 6–8 week old Swiss Webster female mice (Taconic), via intraperitoneal injection of 1×10^6^ parasites. Blood was collected at day 6 (*P. berghei*; >5% parasitemia) and day 9 (*P. yoelii*; >20% parasitemia) of infection.

### Isolation of Infected Erythrocytes and Parasites

*P. yoelii*- and *P. berghei*-infected erythrocytes were obtained using cardiac puncture of mice, washed three times with PBS and separated from white blood cells by centrifugation at 140×*g*. Infected erythrocytes at the schizont stage were isolated at the interphase of a 40% Percoll gradient spun for 15 min at 180×*g*. Blood from uninfected Swiss Webster mice was processed in parallel to obtain control erythrocytes. Mature *P. falciparum*-infected erythrocytes were isolated from the interphase of a 90%/40% Percoll gradient. As a control, uninfected erythrocytes were prepared in parallel. For isolation of parasites from erythrocyte components, synchronized *P. falciparum* trophozoites were isolated from infected erythrocytes as described [29], [31]. Briefly, 1.4–2×10^8^ parasitized erythrocytes were washed twice in PBS, resuspended in 0.05% saponin (Sigma) for 2 minutes at room temperature to remove erythrocyte membranes, and centrifuged at 400×*g* for 5 min. The parasite pellet was washed twice in PBS and stored at −20°C prior to lysis. Control, uninfected erythrocytes were treated similarly.

### Lysate Preparation and Uric Acid Detection

1.4–5×10^8^ infected or uninfected erythrocytes were lysed under hypotonic conditions, using sterile water and reconstituted to 1× PBS post-lysis. Lysates were subjected to ultracentrifugation at 85, 220×*g* for 30 min, and supernatants were collected. The pellets were washed with PBS, spun again, and resuspended in a similar volume of PBS. The pellet resuspension was transferred onto MACS magnetic MS cell separation columns, and flow-through pellet fractions were collected. The columns were washed with PBS, and the hemozoin and eluate fractions were collected in PBS. Uric acid levels were quantified using the Quantichrom™ Uric Acid Assay Kit (Bioassay Systems, colorimetric method). For uric acid inhibition studies, lysate fractions were treated using 0.4 mg/mL uricase from *Candida utilis* (Affymetrix; specific activity: 11.4 units/mg protein). Uricase was purified using an EndoClean endotoxin removal kit (BioVintage). Addition of uricase alone to human dendritic cells did not induce immune activation. Lysate fractions were also treated with 4 units of DNase (TURBO DNase, Life Technologies), or mock treated with a similar volume of PBS. All treatments were incubated at 37°C for 16 h, and kept sterile.

### MSU Preparation

MSU crystals were prepared using previously described methods with modifications. Briefly, a boiling monosodium urate solution (pH 7.5) was prepared by dissolution of equimolar quantities of uric acid and sodium hydroxide (0.03 M) and filtered through a Versapor 3 µM membrane filter (Pall). Sodium chloride (0.1 M final concentration) was added to speed up and improve the uniformity of the crystallization. All crystals were kept sterile, washed with ethanol, dried, autoclaved and resuspended in PBS. MSU samples were sonicated, where indicated, for four 30 sec ultrasonic disintegration cycles at 120W, using a cup horn attachment (Misonix Qsonica Q700 Cell Disruptor Sonicator).

### Dendritic Cell Enrichment and FACS Analysis

Plasma-depleted whole blood was obtained from healthy volunteers. Blood was layered on top of a Ficoll gradient (GE Healthcare) and centrifuged at 1,000×*g* without breaks. Buffy coats were collected and washed, and any remaining erythrocytes were lysed with an erythrocyte lysis buffer (0.15 M NH_4_Cl, 10 mM KHCO_3_, 0.1 mM Na_2_EDTA). Human dendritic cells were enriched from whole PBMCs using the Dynabeads Human Dendritic Cells Enrichment kit (Invitrogen), which enriches dendritic cells by depletion of T cells, B cells, monocytes/macrophages, granulocytes and NK cells.

For cell surface immunostaining, ∼1.5×10^5^ dendritic cells were washed with cold PBS. Cells were then blocked for 15 min at 4°C using human FcR blocking reagent (Miltenyi Biotec), and incubated with the indicated conjugated primary antibodies (BD Biosciences) for 15 min at 4°C. The following primary antibodies were used: anti-CD11c (APC), anti-CD80 (FITC), anti-CD86 (PE) and anti-HLA-DR (PerCP) to mark surface proteins, and for gating of dendritic cells. Flow cytometry was performed on >10,000 events/sample on FACSCalibur (BectonDickinson, San Jose, CA), using CellQuest (Becton Dickinson) software for acquisition and FlowJo (Tree Star) software for analysis. Flow cytometry experiments represent three or more independent experiments with similar fold differences in mean fluorescence intensity (MFI) values.

### Statistical Analyses

Statistical analyses were performed using one-way analysis of variance (ANOVA) with Fisher’s post hoc test for comparison of several means (dendritic cell data; Figure 5). OriginLab software was used and values of p<0.05 were considered significant. For comparison of two conditions (Figures 1–4; S3), a Student’s t-test was used and values of p<0.05 were considered significant. For these data, we show mean ± SEM, from ≥3 independent experiments.

### Dendritic Cell Co-cultures

Dendritic cells were resuspended in RPMI 1640 (Mediatech), supplemented with 10% pooled human AB serum (Valley Biomedicals), 100 mM HEPES (Gibco), and 50 µg/mL gentamicin (Gibco), and seeded in 96-well plates (1.5×10^5^ per well). Stimulation with *P. falciparum* and uninfected erythrocyte lysate fractions for 42 h in the atmospheric conditions of 37°C, 5% carbon dioxide was performed using a 1∶40 dendritic cell to either iRBC or RBC ratio. LPS (Sigma lipopolysaccharides from *Escherichia coli* O26:B6; 1.3 µg/mL) and sonicated MSU (150 µg/10^6^ cells) were used as additional controls.

### Immunofluorescence Assay and Preabsorption of Antibodies

Erythrocytes were adhered to poly-L-lysine coated coverslips in PBS for 30 min at 37°C. Cells were then fixed with 2% paraformaldehyde/0.01% glutaraldehyde for 10 min, and permeabilized for 10 min in 0.125% Triton X-100 (Sigma-Aldrich) in PBS. Cells were blocked for 1 h in 10% goat serum, 100 mM glycine and 0.05% sodium azide in PBS (no BSA was used). The following primary antibodies were used: rabbit anti-uric acid (1∶1500, Advanced Targeting Systems; Figures 1–4, 6, S1–2), rabbit anti-uric acid (1∶1500, Abcam; Figure S2) and rabbit anti-Giantin (1∶1500, Abcam; Figure S1), incubated at 4°C overnight in blocking solution. After washing with PBS, secondary antibodies diluted in blocking solution at 1∶1000 (goat anti-rabbit Cy3, Jackson ImmunoResearch) were added for 1 h at room temperature. Nuclei were labeled with 4′-6-diamidine-2-phenyl indole (DAPI) and coverslips were mounted using DAKO antifade solution. Images were captured with a 100X oil objective lens on an IX70 epifluorescence microscope (Olympus), using similar fluorescence intensities. Images were processed in Adobe Creative Suite, and the ImageJ platform was used to quantify the number of cells with uric acid precipitates (Figure 4E, using a 5 µm background subtraction value and a 30 µm threshold value). It is important to note that soluble uric acid, which is very abundant in erythrocytes (Overgaard-Hansen and Lassen, 1959), is not stained in this assay. This is probably because the fixative agents used, paraformaldehyde and glutaraldehyde, which crosslink primary amines, do not effectively fix soluble uric acid [70], [71]. MSU resuspended in blocking solution (1 mg/mL) was used to preabsorb primary rabbit antibodies overnight at 4°C, where indicated.

### Field Study Site and Sample Collection

Peripheral blood human samples were from malaria patients that attended Laboratorio de Investigaciones de Productos Naturales y Antiparasitarios, Universidad Nacional de la Amazonia Peruana in Iquitos, Peru in July, 2010. The study sites are two networks of communities near Iquitos, the capital of the Amazon province of Loreto, Peru. Patients with fevers ≥37.5°C, or who had experienced fevers within 2 days, were analyzed for parasites in blood smears using light microscopy. If a slide was positive for parasites a nurse and physician would then draw one 8 ml Vacutainer of blood prior to treatment. A 500 µl aliquot of each of these samples was processed for staining of uric acid precipitates as described above.

### Immunoelectron Microscopy

Preparations of mixed blood stages of *P. falciparum* were fixed in 4% paraformaldehyde (Electron Microscopy Sciences, PA) in 0.25 M HEPES (pH 7.4) for 1 h at room temperature, then in 8% paraformaldehyde in the same buffer overnight at 4°C. They were infiltrated, frozen and sectioned as previously described [72]. The sections were immunolabeled with rabbit anti-uric acid antibodies (1∶200 in PBS/1% fish skin gelatin; Advanced Targeting Systems), then with anti-IgG antibodies, followed by 10 nm protein A-gold particles (Department of Cell Biology, Medical School, Utrecht University, the Netherlands) before examination with a Philips CM120 Electron Microscope (Eindhoven, the Netherlands) under 80 kV.

## Supporting Information

Figure S1 **Uric acid antibody preabsorption with MSU reduces antigen recognition in** ***P. falciparum*** **-infected red blood cells.** (A) *P. falciparum*-infected schizonts exhibit uric acid precipitates (red) labeled with anti-uric acid antibodies (Advanced Targeting Systems). (B) Preabsorption of anti-uric acid antibodies (Uric acid preab) with synthetic MSU crystals reduces antibody reactivity. (C) *P. falciparum*-infected schizonts were incubated with anti-Giantin antibodies (Abcam), which label Golgi. (D) Preabsorption of anti-Giantin antibodies with synthetic MSU crystals did not significantly affect antibody reactivity. DAPI labels nuclei (blue). (E) Uninfected red blood cells incubated with anti-uric acid antibodies show no detection of intracellular uric acid precipitates. Bar is 5 µm. These results are representative of three independent experiments.
(TIF)

Figure S2 **Uric acid antibodies from two independent sources detect uric acid precipitates in** ***P. falciparum*** **.** Uric acid antibodies from two independent sources, (A) Advanced Targeting Systems (ATS) and (B) Abcam, detect similar patterns of uric acid immunolocalization (red) in *P. falciparum* schizonts. This pattern of uric acid localization is representative of three independent experiments. DAPI (blue) labels nuclei. Bar is 5 µm.
(TIF)

Figure S3 **Hypoxanthine levels in** ***P. falciparum*** **culture media.** Hypoxanthine levels in *P. falciparum* culture media without exogenous hypoxanthine supplementation were tested before (complete media [CM]) and 24 h after exchange of the culture media in synchronous cultures of ring stage (CM Rings) and trophozoite stage (CM Trophs) parasites. Culture synchronization was performed using a gelatin flotation assay and >90% of the culture media was exchanged daily. Hypoxanthine levels were determined using the Amplex Red Xanthine/Xanthine Oxidase Assay kit (Life Technologies).
(TIF)

Figure S4 **Uricase treatment of purified** ***P. falciparum*** **schizont pellet fractions reduces the stimulatory effect of uric acid on human dendritic cells.** Dendritic cells enriched from 4 independent human donors (D1–4) of peripheral blood mononuclear cells were co-cultured with *P. falciparum* pellet fractions for 42 h. RBC pellet fractions were used as control. Surface expression levels of CD11c, HLA-DR, CD80 and CD86 were analyzed in dendritic cells gated for HLA-DR+. Dendritic cells were cultured alone (grey filled lines), and co-cultured with the following fractions: purified iRBC pellet (red), iRBC pellet treated with uricase (green), uninfected RBC pellet (dark blue) and uninfected RBC pellet treated with uricase (light blue).
(TIF)
