# Supplementary figures and images for: Plasmodium falciparum-Derived Uric Acid Precipitates Induce Maturation of Dendritic Cells
Source: PLoS One. 2013 Feb 6;8(2):e55584. doi: 10.1371/journal.pone.0055584 (PMC3565962; doi:10.1371/journal.pone.0055584)

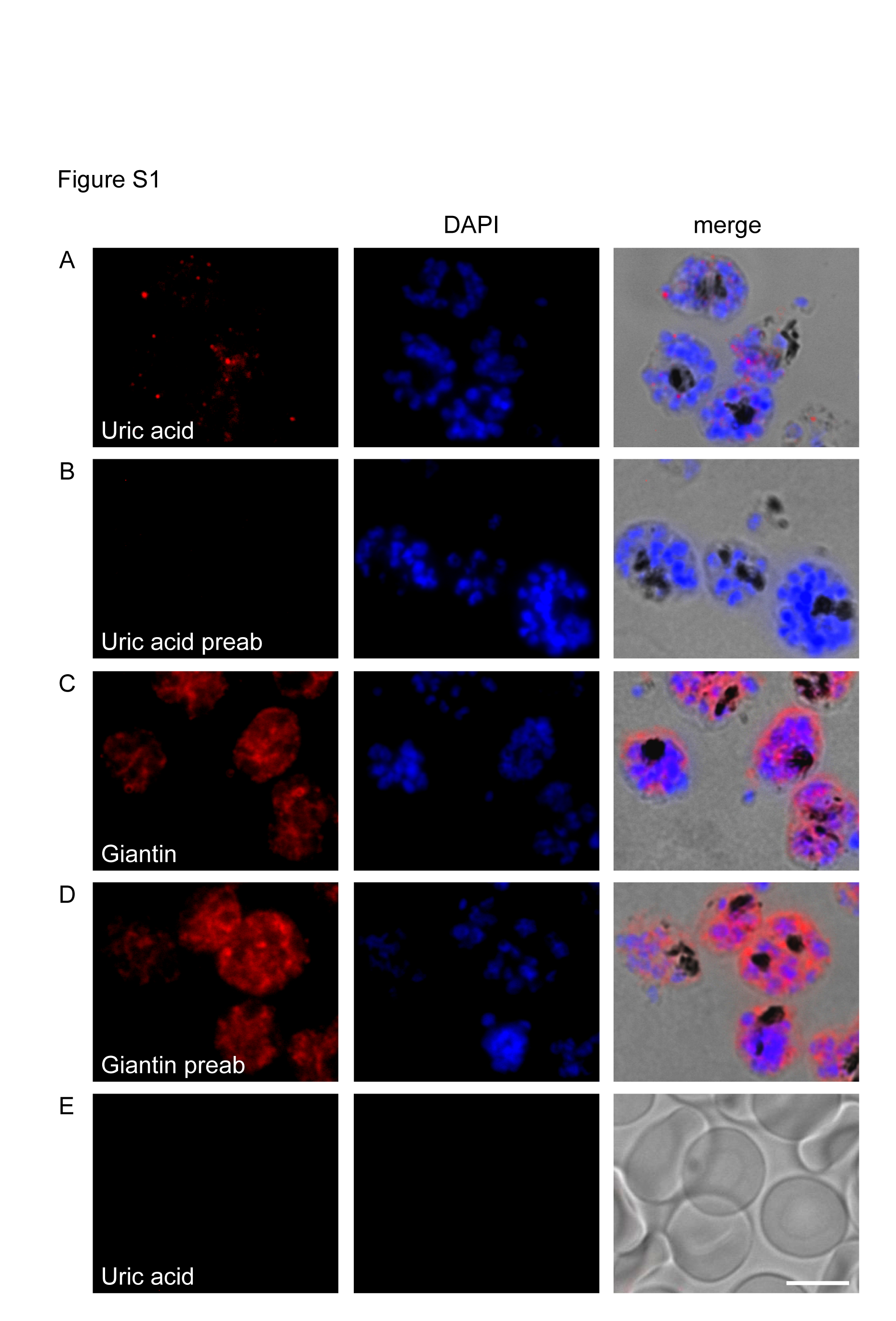

Supplement: Figure S1 — Uric acid antibody preabsorption with MSU reduces antigen recognition in P. falciparum -infected red blood cells. (A) P. falciparum-infected schizonts exhibit uric acid precipitates (red) labeled with anti-uric acid antibodies (Advanced Targeting Systems). (B) Preabsorption of anti-uric acid antibodies (Uric acid preab) with synthetic MSU crystals reduces antibody reactivity. (C) P. falciparum-infected schizonts were incubated with anti-Giantin antibodies (Abcam), which label Golgi. (D) Preabsorption of anti-Giantin antibodies with synthetic MSU crystals did not significantly affect antibody reactivity. DAPI labels nuclei (blue). (E) Uninfected red blood cells incubated with anti-uric acid antibodies show no detection of intracellular uric acid precipitates. Bar is 5 µm. These results are representative of three independent experiments. (TIF) [file pone.0055584.s001.tif]

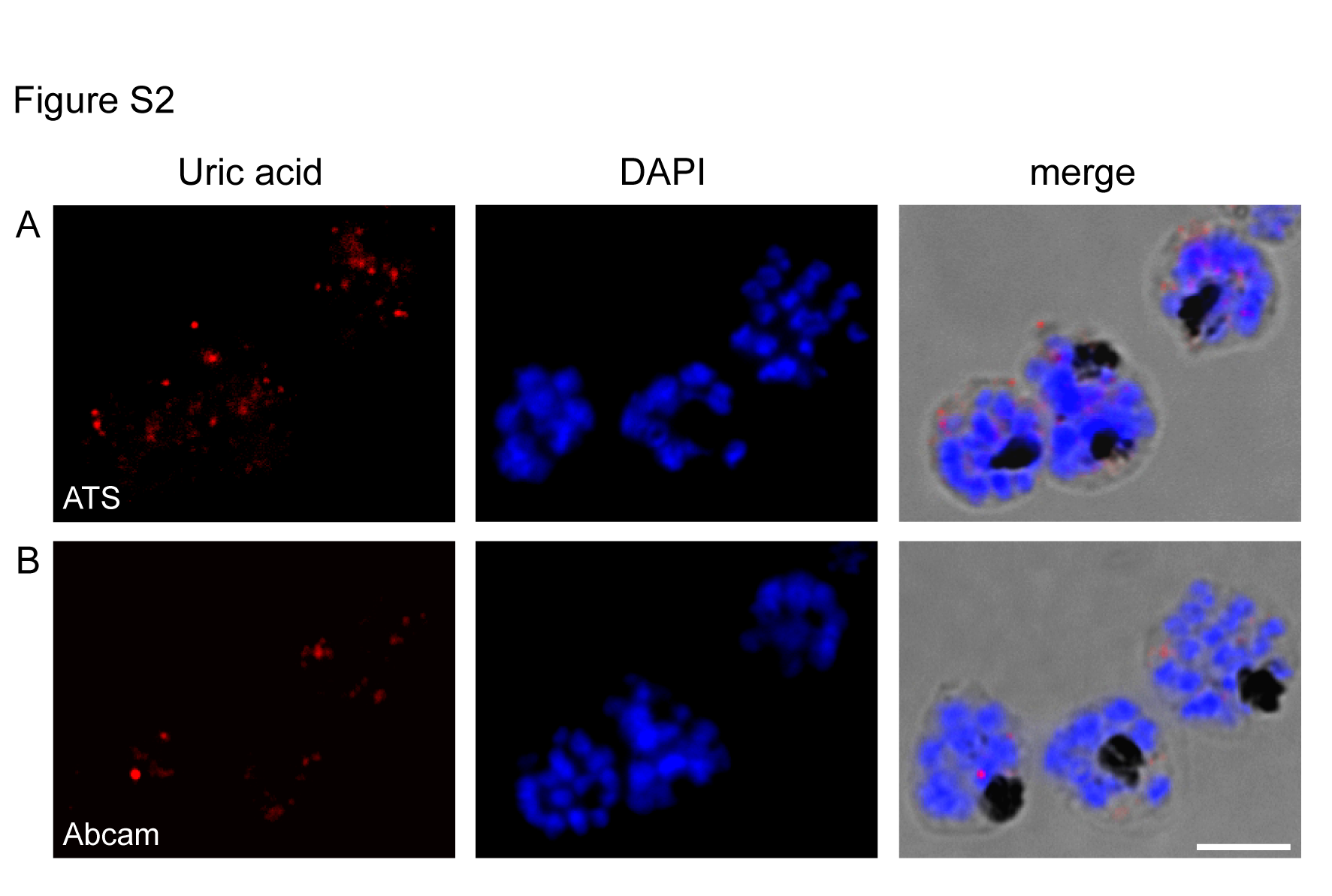

Supplement: Figure S2 — Uric acid antibodies from two independent sources detect uric acid precipitates in P. falciparum . Uric acid antibodies from two independent sources, (A) Advanced Targeting Systems (ATS) and (B) Abcam, detect similar patterns of uric acid immunolocalization (red) in P. falciparum schizonts. This pattern of uric acid localization is representative of three independent experiments. DAPI (blue) labels nuclei. Bar is 5 µm. (TIF) [file pone.0055584.s002.tif]

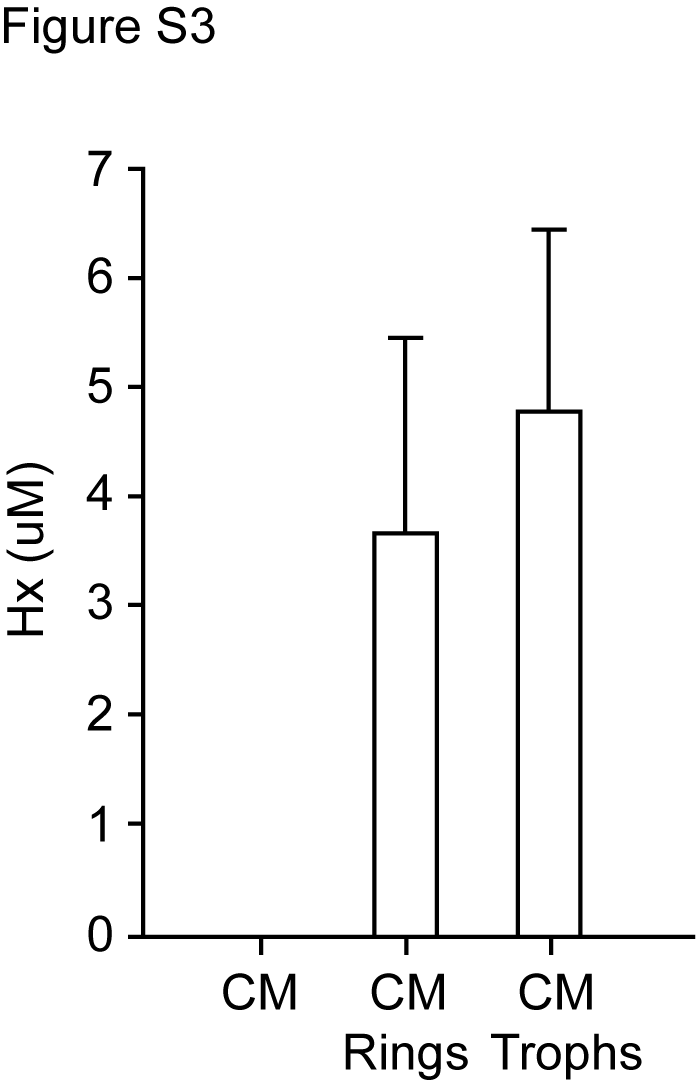

Supplement: Figure S3 — Hypoxanthine levels in P. falciparum culture media. Hypoxanthine levels in P. falciparum culture media without exogenous hypoxanthine supplementation were tested before (complete media [CM]) and 24 h after exchange of the culture media in synchronous cultures of ring stage (CM Rings) and trophozoite stage (CM Trophs) parasites. Culture synchronization was performed using a gelatin flotation assay and >90% of the culture media was exchanged daily. Hypoxanthine levels were determined using the Amplex Red Xanthine/Xanthine Oxidase Assay kit (Life Technologies). (TIF) [file pone.0055584.s003.tif]

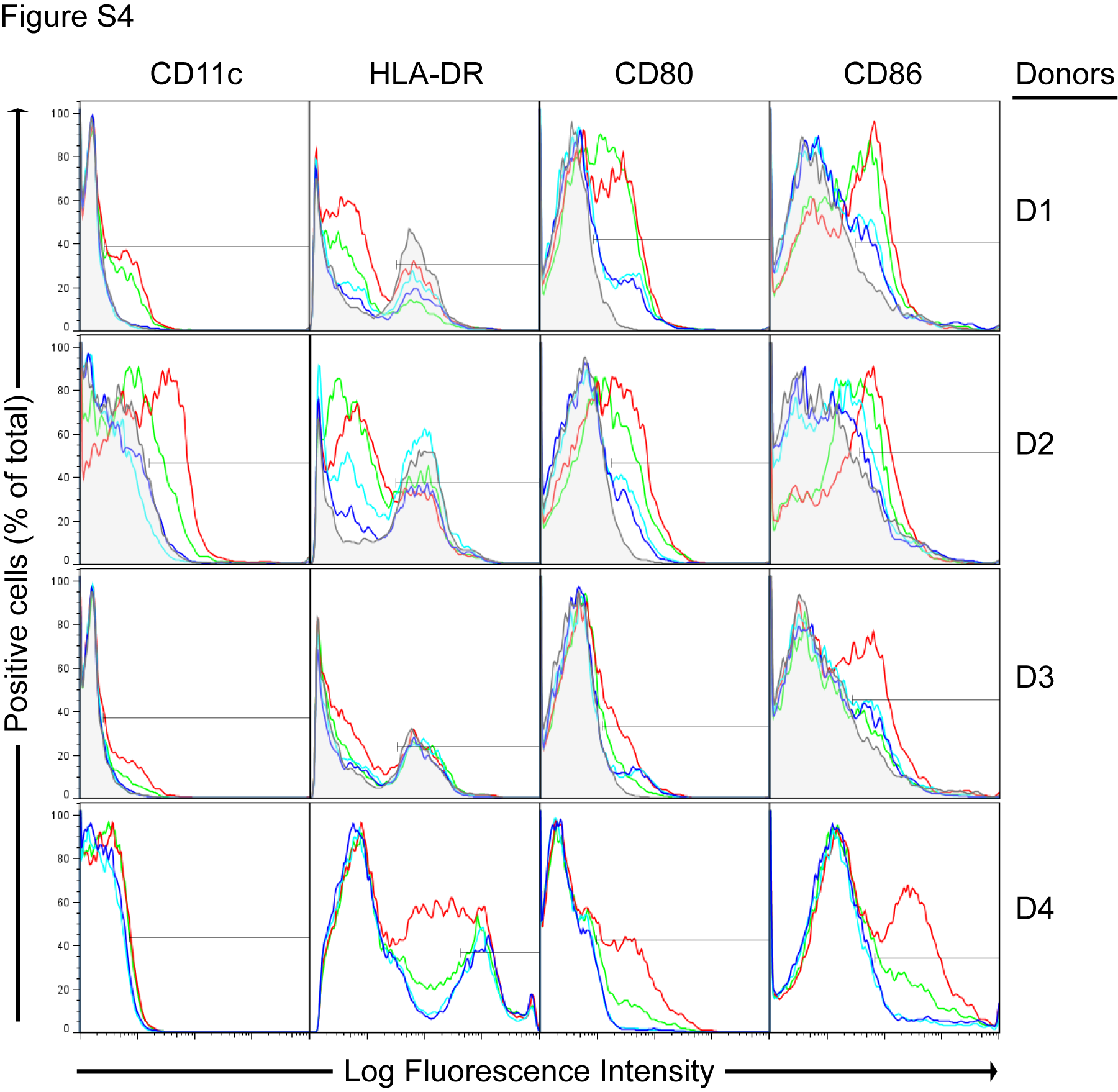

Supplement: Figure S4 — Uricase treatment of purified P. falciparum schizont pellet fractions reduces the stimulatory effect of uric acid on human dendritic cells. Dendritic cells enriched from 4 independent human donors (D1–4) of peripheral blood mononuclear cells were co-cultured with P. falciparum pellet fractions for 42 h. RBC pellet fractions were used as control. Surface expression levels of CD11c, HLA-DR, CD80 and CD86 were analyzed in dendritic cells gated for HLA-DR+. Dendritic cells were cultured alone (grey filled lines), and co-cultured with the following fractions: purified iRBC pellet (red), iRBC pellet treated with uricase (green), uninfected RBC pellet (dark blue) and uninfected RBC pellet treated with uricase (light blue). (TIF) [file pone.0055584.s004.tif]
